# Low-Field NMR Analysis of Chicken Patties Prepared with Woody Breast Meat and Implications to Meat Quality

**DOI:** 10.3390/foods10102499

**Published:** 2021-10-18

**Authors:** Xiao Sun, Jinjie You, Yan Dong, Ligen Xu, Clay J. Maynard, Casey M. Owens

**Affiliations:** 1 School of Biological Science and Food Engineering, Chuzhou University, Chuzhou 239000, China; youjinjienbu@163.com (J.Y.); dongyan_bio@126.com (Y.D.); xlg17855804562@163.com (L.X.); 2 Department of Poultry Science, University of Arkansas, Fayetteville, AR 72701, USA; cjm019@uark.edu (C.J.M.); cmowens@uark.edu (C.M.O.)

**Keywords:** woody breast (WB), chicken patties, low-field NMR, meat quality, texture profile analysis

## Abstract

The scope of this paper was to investigate the effects of water distribution differences on the quality and feasibility of chicken patties supplemented with woody breast (WB). Chicken patties, containing differing amounts of WB (0%, 25%, 50%, 75%, 100%) were analyzed using low-field NMR. Quality differences between chicken patties were further evaluated by combining lipid and protein properties, fry loss (FL), color (L*, a*, b*), texture (hardness, springiness, chewiness, cohesiveness, resilience), microstructure, and sensory characteristics. The results expressed that both lipid and protein oxidation increased and immobilized water in chicken patties can be converted to free water more easily with increasing levels of WB. Additionally, the free water ratio decreased, water freedom increased, and the bound water ratio increased (*p* < 0.05). Fry loss, color, texture (hardness, springiness, chewiness), microstructure, and sensory (character, organization, taste) characteristics deteriorated significantly when the WB inclusion level exceeded 25%. Particularly, characteristics of texture (chewiness and character) and sensory (character and organization) decreased significantly as WB inclusion increased past 25% (*p* < 0.01). Furthermore, fry loss, texture, and overall microstructure partially confirmed the moisture variation of chicken patties as the potential cause of the abnormal quality. Although the experimental data expressed that mixing to 35% WB inclusion was feasible, the practical and economic impact recommends inclusion levels to not exceed 30%.

## 1. Introduction

Woody breast (WB) is an abnormal chicken meat quality problem and an urgent problem that the global poultry industry needs to solve. Currently, researchers believe that the continuous reduction in the broiler growth cycle is a principal factor resulting in the WB condition [1,2]. The primary characterization of WB is that hardness is abnormal, and this is accompanied by the change of tissue morphology and physiochemical properties resulting in worse sensory and processing characteristics than normal breast fillets [3,4]. Severe WB has been known to produce an out-bulging “ridge” in the caudal region, a predominate lignified texture, and petechial hemorrhaging on the surface. Generally, protein composition is reduced, connective tissue is increased, and insoluble collagen content increases in the most severe WB cases [4,5]. Differences in histology, biochemistry, and nutritional composition are the main reasons for low water-holding capacity (WHC), high cooking loss, and food sensory difference in affected fillets [4,5,6]. With these limitations in production use and efficiency, affected WB fillets are still safe and wholesome to consume, with limited quality value, when compared to normal fillets [7,8]. Currently, the mechanism of WB is not well understood. Woody breast is then a challenging issue in poultry production for developing further processed products to comprehensively reduce economic losses.

Meat gels are considered an important means for improving whole meat processing. In a previous study by Zhang [9], the authors found that varying parameters of WB gel change in the ground meat state, which provide a fundamental direction for further processing. Prior to this, there have been attempts to develop and evaluate poultry products using WB meat such as chicken patties and ground sausage [10,11]. Evidence has suggested that the final product quality decreases as WB meat is conjoined to normal breast meat in formed products (chicken patties and meatballs) [12,13]. However, the application of WB in chicken products provide many factors that need to be considered, including the addition ratio of WB, the size of chopped and mixed grain, and the addition of other auxiliary materials [14]. Furthermore, the determination of the optimal inclusion of WB to maintain the quality of chicken patties is key to solving the economic loss of discarding WB during poultry meat processing. Therefore, it is important to investigate quality characteristics of chicken patties based on the amount of WB inclusion and determine a suitable inclusion rate in chicken patties.

The application of low-field NMR in meat quality analysis is mainly to explore the proton distribution and density through ^1^HNMR (nuclear magnetic resonance hydrogen spectroscopy), as to reflect the quality of meat products [5,6]. Low-field NMR has increased in poultry research to further explore variation in WB quality by assessing microstructure composition. Currently, characteristics of water in the WB condition have been analyzed by low-field NMR, and the results show that the content of free water in WB was significantly higher than that in normal meat [6,15]. Although the application of low-field NMR in WB has been common, the quality evaluation of WB in further processed products has not been extensively reported [16]. Therefore, the current study focused on assessing the relationship between water distribution and other meat quality parameters by low-field NMR in chicken meat patties. Overall, inclusion levels of WB (0%, 25%, 50%, 75%, 100%) were targeted and prepared to assess intermediate differences in formulation methods. The feasibility of adding WB and setting an optimal quantity range were determined by index analysis of lipid and protein properties, fry loss (FL), low-field NMR, color, texture, microstructure, and sensory evaluation.

## 2. Materials and Methods

### 2.1. Sample Preparation

Intact whole breast fillets were collected from a broiler processing line, which were divided into normal (NORM, 7.5 kg) and severe (SEV, 7.5 kg) categories according to the subjective scale proposed by Tijare [17] with modifications provided by Sun [18]. All fillets in the NORM category had a soft and smooth appearance, good flexibility throughout and hung naturally when laid across the hand, whereas all SEV samples were hard/firm to the touch and conveyed ridges along the caudal region. All breast fillets were encapsulated in individual food grade bags and then stored at 4 °C for further experimental analysis. Surface fat, muscle fascia, and exterior connective tissue of each fillet was removed. The NORM and SEV chicken breasts were ground separately (3 min) in a precooled mincer (Knife Mill Grindomix GM 200, Retsch, Haan, Germany) at 3500 rpm. Formulation was set to include WB at 0%, 25%, 50%, 75% and 100% with the remaining levels completed with NORM fillets. According to the defined inclusion rates of WB (0%, 25%, 50%, 75%, 100%), the ground meat from each severity was mixed to obtain 2 kg of minced meat for each treatment, which was refrigerated at 4 °C for later use. Furthermore, minced breast meat for each treatment and pork fat (purchased from local supermarket of Chuzhou) were mixed according to a 9:1 ratio. Formulation also included 2% starch, 2% soy protein, and 1% salt. After fully mixing, 100 g (m_a_) of chicken paste was taken and pressed into round chicken patties with a 10 mm thickness and 100 mm diameter molding machine. In this study, a total of 60 chicken patties (*n* = 12/treatment) was considered for further quality analysis. The round chicken patties were submerged in oil with a temperature setting of 160 °C and removed when the internal temperature of each chicken patty met or exceeded 76 °C. After being removed, patties were allowed to drip dry of excess oil on a towel covered baking sheet and then cooled to room temperature.

### 2.2. Determination of TBARS Value

TBARS value determination was based on the procedure provided by Zhang [19] and modified to some extent. Each ground chicken patty sample (4 g) was added into 20 mL of trichloroacetic acid solution (0.2 g/mL, containing 0.1% EDTA·2Na) and homogenized in duplicate at 7500 rpm for 30 s. Samples were then centrifuged at 12,000× *g*, held at 4 °C for 5 min, and 5 mL of supernatant was collected in a colorimetric tube. Following collection, the same volume of thiobarbituric acid solution (20 mmol/L) was added back into the tube. The mixture was heat treated in a boiling water bath (100 °C) for 30 min, cooled to room temperature, and absorbance was measured at the 532 nm wavelength.

### 2.3. Protein Oxidation Analysis

#### 2.3.1. Determination of Sulfhydryl Groups and Surface Hydrophobicity

Myofibrillar proteins (MP) from chicken patties with different WB inclusion were extracted according to Han [20] with minor modifications. To begin, the initial mass of minced meat was weighed and multiplied by 4 to determine the volume of standard salt solution to add (0.1 M KCl, 20 mM K_2_HPO_4_, 20 mM KH_2_PO_4_, 1 mM EGTA, 2 mM MgCl_2_·6H_2_O, pH 7.0, 4 °C). Samples were homogenized utilizing an ice bath method at 7000 rpm for 30 s. Following homogenization, two layers of medical gauze were used to filter impurities. With impurities removed, centrifugation was performed (4 °C, 2000× *g*, 10 min), supernatant discarded upon completion, and re-centrifuged three subsequent times. Then, four times the volume of the product of 0.1 M KCl solution was added to the precipitate and homogenized in a 7000 rpm ice bath for 30 s. Centrifugation was repeated twice (4 °C, 2000× *g*, 10 min). The centrifuged product was considered pure MP which were then dissolved in PBS (0.6 M NaCl, 20 mM K_2_HPO_4_, 20 mM KH_2_PO_4_). For this procedure, BSA was used as the standard protein and its concentration was determined by the biuret method.

The determination of reactive sulfhydryl content (R-SH) was based on the method provided by Guo [21]. A volume of 5 mL of myofibrillar protein solution (1 mg/mL) was thoroughly mixed with 20 µL 5,5’-dithio-bis-2-nitrobenzoic acid (DTNB) and left at room temperature for 1 h. Product absorbance was measured at 412 nm on a microplate reader.

Surface hydrophobicity of MP was determined utilizing the probe binding method [22], with ANS (8-aniline-1-naphthalene sulfonic acid) as a fluorescent probe. To begin, 20 μL of ANS solution (15 mmol/L ANS, pH 7.0) was added to 4 mL of a MP sample (1.0 mg/mL). The samples were thoroughly mixed and incubated at dark room temperature for 20 min. The fluorescence intensity of the samples was measured at an excitation wavelength of 375 nm and an emission wavelength of 385–535 nm using a microplate reader.

#### 2.3.2. Sodium Dodecyl Sulfate-Polyacrylamide Gel Electrophoresis (SDS-PAGE)

SDS-PAGE procedures were carried out according to the method presented in Li [23]. The original 2.3.1 MP solution was utilized and adjusted to the concentration of 1 mg/mL, mixed with a 30 μL protein sample containing 10 μL 4×DTT SDS-PAGE sample buffer to create a final sampling buffer. This sample loading buffer was heated in a water bath at 100 °C for 10 min to denature proteins. A volume of 10 μL of the sample loading buffer then contained 5 μL molecular standard marker (Thermo Fisher Scientific Co., Ltd., Shanghai, China) and 5 μL of 4–20% precast gel lane (GenScript, 12% polyacrylamide, 15 wells). A MiniProtean electrophoresis apparatus (Bio-Rad Laboratories, Hercules, CA, USA) was used at 4 °C with the following voltage: 80 V for 20 min and 100 V for 80 min. Thereafter, the gel was stained for 40 min and decolored for 10 h by hand using a staining solution and decolorizing liquid (GenScript Biotech Corp, Nanjing, China). The gels were scanned using a molecular imaging system (Gel Doc XR+, Bio-Rad Laboratories, Hercules, CA, USA).

### 2.4. Fry Loss

Three patties were prepared for each treatment (0%, 25%, 50%, 75% and 100%), fried, and then cooled to room temperature. Chicken patties were weighed again when the liquid oil visually present on the surface was completely dried and recorded as m_b_. Fry Loss (FL) was calculated by the percentage change of the weight prior to frying.

$$\mathrm{FL}/\%=\frac{m_{a}-m_{b}}{m_{a}}\times 100\%$$

Equation: m_a_ indicates the mass of chicken patties prior to frying; m_b_ indicates the mass of fried chicken patties.

### 2.5. Nuclear Magnetic Resonance (NMR) Relaxation Measurements

Water state distribution of prepared chicken patties was measured using a Niumag Pulsed NMR analyzer. Parameter settings targeted the method of Tasoniero et al. [6], with some modifications. The cube (10 mm × 5 mm × 8 mm) shaped sample was sliced from each gradient treatment and placed into a 60 mm-diameter clear nuclear magnetic tube. According to the sequences provided by Carr-Purcell-Meiboom-Gill (CPMG), the main frequency and offset frequencies of the low-field NMR measurements were set to 25 Hz and 411,671.61 MHz, respectively. Sample readings were collected 104,020 times with 8 repeating scans and a sampling frequency of 200 kHz. T_2_ measurements were collected based on a τ-value of 300 μs and repeated 32 times. Data presented as the relaxation times of T_2_ parameters in hydration water (T_2b_, 0.1–10 ms), immobilized water (T_21_, 10–100 ms), free water (T_22_, 100–1000 ms), time constants of each, relative areas or proportion of each water frequency, and low-field NMR inversion images.

### 2.6. Color of Patties

The color of cooked chicken patties (CIE L*, a*, b*) was measured using a portable Minolta CR-400 (illuminate D and 65° standard observer) device (Minolta Camera Co., Osaka, Japan). The color was measured on the external surface and chicken patties were cut along the center line to measure the interior color. All measurements were carried out on section areas of meat cubes. Prior to color analysis, the instrument was standardized using a white calibration plate (CIE L* = 96.86, CIE a* = −0.15, CIE b* = 1.87; white board, no. 20933026). Three individual color readings were collected for each sample. At the completion of the third sample collection, all three values were averaged and recorded [24].

### 2.7. Texture Profile Analysis (TPA)

Three chicken patties were prepared per treatment, each sample was cut into 6 meat cubes (8 mm × 8 mm × 8 mm) from the 30 mm radius of the center circular area. The texture profile attributes (TPA) were determined using a texture analyzer (TA-XT. plus, Stable Micro system Ltd., Surry, UK) equipped with a cylindrical probe (P/36R). TPA settings were set to match those presented in Mudalal [4] with the following modifications: test speed was 1 mm/s, pre-test speed was 4 mm/s, post-test speed was 4 mm/s, strain was set at 30%, and trigger force was set to 5 g. TPA measurements were collected in duplicate for each sample, and the average value of hardness, springiness, chewiness, cohesiveness and resilience were recorded.

### 2.8. Microstructural of Patties

Meat cubes (measuring approximately 5 × 5 × 5 mm) were cut from each chicken patty within the central area radius of 30 mm. Microstructural samples (10 μm thick) were prepared using a microtome (Sakura Finetek USA, Inc., Leica, Germany) and transferred onto fluorescence microscope slides. Under microscopic observation with 100× magnification, sample images were captured and recorded using the Carl Zeiss microimaging system (Carl Zeiss, Gottingen, Germany).

### 2.9. Sensory Evaluation

Sensory evaluation of chicken patties was analyzed using the method by Morita [25] with slight modifications according to sensory parameters. Ten professional sensory scientists (five male and five female) were selected to evaluate and describe sensory samples. The sensory evaluation team was trained by the procedures in Zhuang [26], training panelist on texture attributes (hardness, cohesiveness) and flavor attributes (flavor, salt) before sensory evaluation was conducted. Each professional was asked to rinse their mouth with deionized water 2–3 times before and after each test. The character, organization, and taste of each chicken patty was rated on a scoring scale of 0–100 (shown in Table 1) with description and grades according to Zhuang [26]. The total sensory score (TSE) of each sample was calculated as 40% character score, 30% organization score, and 30% taste score.

### 2.10. Optimal Application of WB

Gradient experiments were carried out using the optimal additive range determined by the above experiments with ±5% gradient difference and 0% and 100% set as the control groups. The optimal additive quantity was determined by the presence of quality indicators with significant differences which were carried out in the above experiments. Sample preparation of chicken patties is the same as described in the above experiment.

### 2.11. Data Analysis

Meat quality measurements (TBARS, R-SH, FL, color, texture attributes of TPA), water distribution properties (time constants of T_2b_, T_21_ and T_22_), and sensory evaluation data were analyzed using one-way Analysis of Variance (ANOVA) of SPSS 20.0 with measured results expressed as mean ± standard deviation. The main effect in this study was to evaluate the inclusion rate of WB in chicken patties (0%, 25%, 50%, 75%, 100%). Where appropriate, means were separated using Tukey’s honest significant difference test and the significance level was set at *p* ≤ 0.05.

## 3. Results

### 3.1. Lipid Oxidation

**Figure 1:**
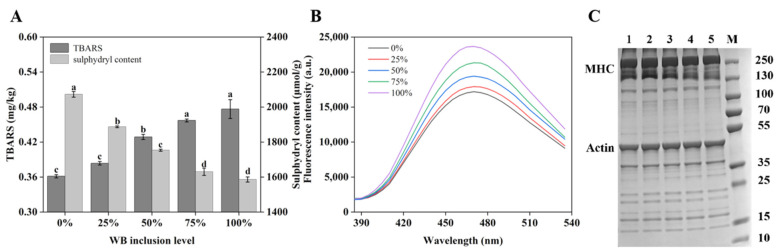
TBARS, reactive sulfhydryl content (**A**), surface hydrophobicity (**B**), and SDS-PAGE patterns from MP (**C**) in chicken patties with varying woody breast inclusion levels. M represents the designated molecular weight marker. Lines 1–5 indicate the woody breast inclusion levels of 0–100% with 25% increasing steps, respectively. MHC: myosin heavy chain. ^a–d^ means not sharing a common letter are considered significantly different (*p* < 0.05).

TBARS values for chicken patties with different amounts of WB are shown in Figure 1A. With the increase of varying WB inclusion levels, TBARS estimates increased gradually (*p* < 0.05). There was no significant difference in TBARS values between 0% and 25% WB inclusion (*p* > 0.05), which were 0.366 and 0.378 mg/kg, respectively. With increasing WB inclusion, TBARS values increased significantly and reached the highest value of 0.474 mg/kg at 100% WB inclusion levels.

### 3.2. Protein Oxidation

As shown in Figure 1A, the reactive sulfhydryl content in myofibrillar proteins (MP) decreased significantly between 0% and 75% inclusion levels (*p* < 0.05). There was no significant difference between the 75% and 100% inclusion level (*p* > 0.05). The maximum reactive sulfhydryl content was 2072.68 ± 16.35 μmol/g at 0% inclusion. The lowest value was 1586.32 ± 14.57 μmol/g at 100% inclusion.

Figure 1B shows the surface hydrophobicity of MP. Fluorescence intensity reflects surface hydrophobicity intensity. As can be seen from Figure 1B, the fluorescence intensity gradually increased with increasing WB, indicating that the surface hydrophobicity of MP increased. The lowest fluorescence intensity was 15,220 when the WB inclusion level was at 0%, and the highest fluorescence intensity was 23,572 when the WB inclusion level was at 100%.

Furthermore, the SDS-PAGE of MP for different WB inclusion levels is presented as Figure 1C. Lines 1, 2, 3, 4, and 5 indicate the WB inclusion rates of 0%, 25%, 50%, 75%, and 100%, respectively, with M as the marker position. Multiple bands appeared at the same position under varying amounts of WB inclusion, indicating similar protein composition. However, increasing WB inclusion changed the color of myosin heavy chain (MHC) band (became lighter), while the color of the actin strip expressed relatively no differences.

### 3.3. Frying Loss

Fry loss results are displayed in Table 2. Fry loss increased (*p* < 0.05) as the content of WB inclusion increased. There were no differences in FL values between chicken patties produced with 0% and 25% WB inclusion (*p* > 0.05), which were 7.80 ± 0.20% and 9.23 ± 0.20%, respectively. Chicken patties containing 50% WB (11.33 ± 0.42%) exhibited higher FL, when compared to that containing 25% WB (9.23 ± 0.20%). However, FL of chicken patties produced by adding 50% WB was significantly lower (*p* < 0.05) than samples containing 100% WB. There were no significant differences in FL between chicken patties containing 75% and 100% WB (*p* > 0.05). The greatest FL was obtained at 100% WB inclusion (13.43 ± 0.80%), which was 1.7 times higher than patties with 0% inclusion (7.80 ± 0.20%).

### 3.4. Low-Field NMR

**Figure 2:**
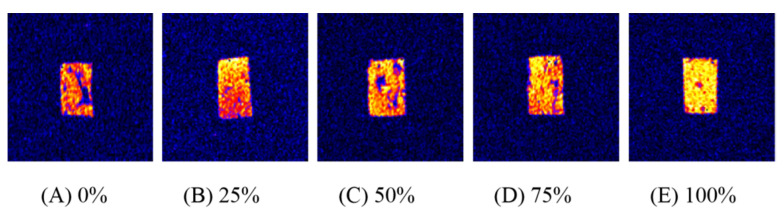
Low-field NMR imaging of chicken patties prepared with different proportions of woody breast (WB) inclusion. (**A**), (**B**), (**C**), (**D**), and (**E**) represent the NMR imaging of chicken patties with 0%, 25%, 50%, 75%, and 100% WB inclusion, respectively.

**Figure 3:**
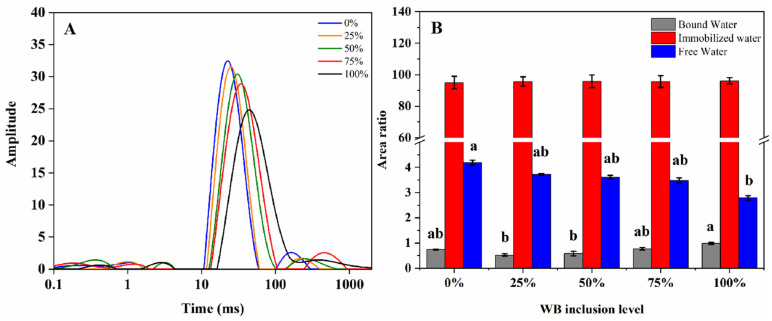
Low-field NMR peak (**A**) and relative area ratio (**B**) of chicken patties with varying woody breast inclusion levels. ^a,b^ means not sharing a common letter are considered significantly different (*p* < 0.05).

Figure 2 provides low-field NMR inversion imaging of chicken patties, where A, B, C, D, and E represent the NMR imaging of chicken patties with 0%, 25%, 50%, 75%, and 100% WB inclusion, respectively. As seen in Figure 2, sample brightness gradually increased as the amount of WB inclusion increased. Figure 3A exhibits inverted chicken patty NMR relaxation measurements with 3~4 peaks, which are represented by T_2b1_ (strong bound water), T_2b2_ (weak bound water), T_21_ (immobilized water), and T_22_ (free water), respectively. As seen in the Figure 3A, T_21_ and T_22_ peak relaxation curves of chicken patties shift to the right with increasing WB inclusion. Sample peak area for patty treatments is expressed in Figure 3B. For this dataset, the peak area ratio refers to the type of water present (bound water, immobilized water, and free water) and the relaxation period (T_2b,_ T_21_, and T_22_) in comparison to the total area of each sample. When assessing bound water, the 100% WB treatment expressed the highest bound water content when compared to all other treatments (25% = 50% < 0% = 75% < 100%, *p* < 0.05). The current data expressed no significant differences for immobilized water for all treatments (*p* > 0.05). However, there was a significant difference observed in free water content between the 0% and 100% treatments (*p* < 0.05).

### 3.5. Color

Data collected for external and internal surface color for chicken patties are expressed in Table 2. The external L* values between the 0%, 25%, and 50% group had no significant difference (*p* > 0.05), which were 63.27 ± 0.75, 62.48 ± 0.47, and 63.85 ± 0.46, respectively. Similarly, there were no significant differences L* values between the 75% and 100% treatments (0%, 25%, and 50%, *p* < 0.01). There was no difference in internal L* between all samples (*p* > 0.05), which were 77.77 ± 1.03, 78.93 ± 0.18, 79.13 ± 0.22, 78.85 ± 0.20, and 77.85 ± 0.76, respectively. Internal L* values for chicken patties were all higher than external L* values (*p* < 0.05). The WB inclusion rate had a significant influence on external a* value (*p* < 0.01). However, low WB inclusion (25%) had no significant effect on external a* (*p* > 0.05). With increasing WB inclusion, the external a* and internal a* gradually decreased. In addition, supplemental increases of WB had a significant effect on both the external and internal b* values of chicken patties. The external b* value of chicken patties significantly decreased, while internal b* values significantly increased. When the supplemental amount of WB inclusion reached 100%, the external and internal b* values were 33.84 ± 0.28 and 15.82 ± 0.07, respectively.

### 3.6. Texture Attributes

Texture properties of chicken patties with different WB additions are shown in Table 3. Hardness, chewiness, and cohesiveness were assessed as objective quality and structure indices of each patty. Likewise, hardness, chewiness, and cohesiveness have significant influence on the sensory evaluation of meat patties. Hardness, springiness, and chewiness were different between WB inclusion rates (*p* < 0.01), while cohesiveness and resilience were similar between treatments (*p* > 0.05). As WB inclusion gradually increased, hardness, springiness, and chewiness significantly decreased (*p* < 0.01). When WB supplementation was 100%, hardness, springiness, and chewiness reached the lowest values, which were 1637.81 g, 0.86, and 1348.76 g, respectively. There was no significant difference (*p* > 0.05) for springiness of chicken patties between the 0% and 25% treatments. When WB inclusion reached 25%, the springiness reached a maximum value of 0.90 ± 0.01. As WB inclusion exceeded 25%, springiness decreased significantly (*p* < 0.01). In addition, there was no significant difference between cohesiveness and resilience on WB supplementation amount (*p* > 0.05).

### 3.7. Microstructural

**Figure 4:**
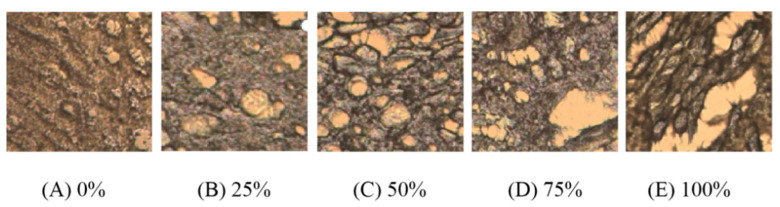
Microstructure images of chicken patties with varying levels of woody breast addition. (**A**), (**B**), (**C**), (**D**), and (**E**) represent the microstructure of 0%, 25%, 50%, 75%, and 100% WB inclusion, respectively.

Illuminated microscopic makeup of chicken patties are exhibited in Figure 4. A, B, C, D, and E represent the microstructure of 0%, 25%, 50%, 75%, and 100% WB inclusion, respectively. In this study, as WB inclusion increased, microscopic structures of chicken patties deteriorated and expressed dilapidation as deep gouges increased throughout. The microstructure of each chicken patty was severely damaged with 100% inclusion, and the polymerization degree declined as the amount of WB increased. In addition, increasing WB inclusion produced a loose, incomplete, and visually detectable reduction in overall structural appearance.

### 3.8. Sensory Attributes

Sensory evaluation attributes for chicken patties with varying levels of WB inclusion are expressed in Table 3. All attributes were different among WB inclusion rates (0%, 25%, 50%, 75%, and 100%, *p* < 0.01). Overall, sensory attributes (character, organization, taste, and total score) were similar between 0% and 25% inclusion, and then significantly decreased as WB inclusion exceed 50%. The worst sensory values were expressed at 100% WB inclusion with character (41.80 ± 5.51), organization (52.80 ± 2.90), taste (49.50 ± 3.36), and score (47.41 ± 2.85). The best sensory values were expressed at 0% or 25% WB inclusion with character (25%, 65.10 ± 4.49), organization (0%, 69.60 ± 2.43), taste (25%, 64.00 ± 3.32), and score (25%, 65.04 ± 2.02).

### 3.9. Optimal WB Application

**Figure 5:**
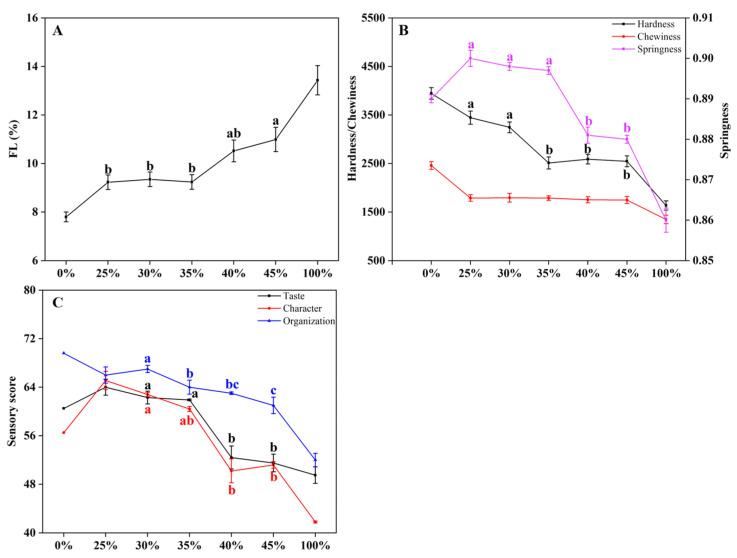
Different indicators of chicken patties prepared with varying levels of woody breast (WB) inclusion. (**A**) Fry loss (FL); (**B**) TPA characteristics (hardness, chewiness, springiness); (**C**) Sensory evaluation scores (taste, character, and organization). ^a,b^ Means within the same indicator followed by different superscript letters differ significantly (*p* < 0.05).

The result of FL, TPA measurements and sensory attributes are expressed in Figure 5. According to the above experimental results, when WB inclusion exceeds 25%, multiple quality parameters show a significant decrease. Striving for the optimal inclusion rate, a verification test was conducted using 25%, 30%, 35%, 40%, and 45% WB inclusion as experimental gradients (0% as the blank group and 100% as the control group). Fry loss, TPA measurements (hardness, springiness, and chewiness), and sensory attributes (character, organization, taste) were selected as indicators for verification. Overall, FL, texture properties (TPA), and sensory evaluations were similar among WB inclusion of 25%, 30%, and 35%. As WB inclusion exceeds 35%, FL of chicken patties increased significantly (*p* < 0.01). Meanwhile, TPA measurements (hardness, springiness, and chewiness) and sensory attributes (character, organization, taste) decreased significantly (*p* < 0.05).

## 4. Discussion

Oxidation is one of the most important non-microbial degradation pathways of meat, which can lead to degradation of color, aroma, flavor, and other sensory characteristics while decreasing the nutritional value of meat products [27]. Figure 1A expresses that TBARS values increase significantly with increasing WB inclusion, indicating that the degree of lipid oxidation gradually increases. Among the lipid oxidation products, aldehydes, ketones, acids, esters, and alcohols generated by the β-fracture of hydroperoxide determine the smell of meat, and excessive lipid oxidation will produce a rotten taste in meat products [28]. Therefore, excessive inclusion of WB can cause lipid peroxidation and lead to the generation of off flavors or even a rotten flavor.

Figure 1A,B shows that with increasing WB inclusion, the content of reactive sulfhydryl content of MP significantly decreased, thus significantly increasing surface hydrophobicity. The results showed that WB inclusion accelerated the oxidation of myofibrin. Research has shown that the fat content of WB meat is higher than normal meat, and previous studies have shown that meat with high fat content has a stronger degree of protein oxidation [29]. The increase of surface hydrophobicity of MP is closely related to the conformation and oxidative degree of each protein. Moreover, the exposure of non-polar amino acids on the surface caused by protein oxidation is the main reason for the increase of surface hydrophobicity [30]. As shown in Figure 1C, MHC bands became shallow with increasing WB inclusion, indicating increased damage to the MHC and further degradation of MP, which was consistent with surface hydrophobicity. The degradation of MP could also be considered as a reason for low nutritional value and poor processing characteristics in WB meat. Therefore, large substitution rates of WB will increase the oxidation degree of MP, creating a decrease in nutritional value and poor sensory texture characteristics of a final product.

Low-field NMR has been successfully used for many years to investigate meat quality and deepen the knowledge about the behavior of water in meat abnormalities such as WB and pale soft exudative (PSE) conditions [5,31]. Data from these previous studies indicate WB fillets have a significant impact on the distribution of water within the muscle tissue and show altered moisture properties (more extra-myofibrillar water, less intra-myofibrillar, and less hydration water) compared to normal fillets [5,6,16]. Figure 2 exhibits that low-field NMR inversion imaging of chicken patties, where A, B, C, D and E represents the low-field NMR imaging of chicken patties with 0%, 25%, 50%, 75%, and 100% of WB addition, respectively. As shown in Figure 2, sample brightness gradually increased with increasing WB inclusion, which indicates an increasing density of hydrogen protons parallel to WB inclusion. Conversely, Figure 3A expresses relaxation times of T_21_ and T_22_ relaxation curves shifting significantly to the right with increasing amounts of WB inclusion. This indicates that the water fluidity increases in chicken patties, and the transition from immobilized water to free water becomes more active [9]. For this reason, water activity is the key factor affecting the quality change during the storage of meat products [16]. In addition, the ratio of the integral area to the total area in the relaxation time T_2_ interval can represent the relative content of hydrogen protons in the interval. Figure 3B exhibits that the free water proportion of chicken patties gradually increased, while the immobilized water proportion decreased, which further verify the results presented in Figure 3A.

Bound water is biologically important for organism survival and viably important in the conversion of muscle to meat. Bound water is the intercellular macromolecule water that can bind to protein and other components in the cell to build a tensile protein network during rigor mortis. The higher the proportion of bound water, the lower the binding affinity to proteins, resulting in difficulties forming a dense protein network structure [32]. Microstructure results for each inclusion rate of WB are exhibited in Figure 4. In the current experiment, high WB inclusion rates were the main reason that protein structures directly affected the breakage and cavity of the molecular microstructure. The lower affinity of water retention in WB results in a reduction in starch binding and other auxiliary materials not fully blending into chicken patties, forming granular bonds after frying to give the unique appearance [12]. Similar results have also been reported in the research by Zhang [9]. In addition, high salt-soluble protein extraction could contribute to protein-protein cross-linking, promoting the formation of more uniform three-dimensional networks [33]. We suppose that bound water further alters the hardness and chewiness of chicken patties by affecting the formation of protein networks. Pietrasik [34] and Sun [11] also found that protein content and the use of WB caused changes in textural parameters such as hardness, chewiness, and springiness of sausage. In Table 3, hardness, springiness, and chewiness decreased significantly with increasing WB inclusion. Previous studies have shown that the hardness of whole meat increased as final WB grade increased. However, Zhang [9] found that the gel properties of WB minced meat were different from that of whole meat, resulting in an opposite trend of hardness change. The authors propose that this result is due to the size of gel granules affecting the content of salt-soluble protein, leading to differences in the gelation properties of meat gels [5,9]. Nevertheless, we found that an appropriate amount of WB inclusion in chicken patties (<25%) had no obvious effects on parameters such as hardness, springiness, and microstructure. This further indicates that the quality of WB in gels was acceptable within a certain range.

Water holding capacity (WHC) is an important index reflecting the quality of meat products [35]. The loss of free water is the main effect causing differences in WHC. Hence, during processing and storage, the content of free water directly impacts final meat quality. Figure 3B exhibits the increasing free water content of patties as WB inclusion increased, indicating that the free water content of WB was higher than that of normal chicken breast meat. Additionally, the quality of high WB inclusion was prone to deterioration during processing and storage, which is consistent with lipid and protein oxidation. Conversely, the processing of frying was adopted in this study as free water can evaporate when in contact with high temperature cooking oil. The large amount of evaporation of free water could potentially be a primary reason for the significant increase in frying loss of chicken patties as WB inclusion increased. A key observation was noted as the loss of free water increased, the patties gradually “puffed up” in nature, and the infiltration of cooking oil was more obvious. This infiltration of cooking oil may have also produced the direct change of color in patties.

The color of cooked meat depends on the relative amount of denatured and non-denatured myoglobin, as well as the degree of lipid peroxidation in the meat products [36]. In this study, the decrease of a* value in chicken patties could potentially be explained by the rapid oxidation of myoglobin to brown ferric myoglobin and taupe globulin to chlorinated hemochromogen [37]. Zhuang [38] also found that the WB myopathy could result in discoloration of cooked meat, resulting in a decrease in lightness of meat products. In this study, decreased b* values of the exterior surface of chicken patties may be due to the inclusion of WB, which is in agreeance with Chatterjee [39]. In this experiment, L* increased as WB inclusion increased, which could be expected as collagen content has been consistently observed in severe WB fillets. Alongside an increasing collagen content, increasing WB inclusion caused excessive lipid and protein oxidation, which could have influenced a shift in L* values. Furthermore, evidence suggests that CIE L* values can be significantly altered in WB products following mass quantities of water loss post cook [20,40,41].

Sensory evaluation has been considered one of the most direct methods to obtain product quality attributes. Previous studies have found that texture has obvious influence on sensory perception. At the same time, this study found that with increasing WB, the lipid and protein oxidation degree of chicken patties increased significantly, which had an important impact on the flavor and taste of chicken patties [27]. Sun [11] analyzed sausages with WB inclusion and found that microstructure, hardness, and chewiness were the main factors affecting taste, organization, and flavor of the products. In the current study, the significant deterioration in the taste of chicken patties could be attributed to the decrease in hardness and chewiness, while the looseness of the microstructure may also reflect differences in tissue structures from normal fillets because of extensive WB inclusion, similar to those previously described [11,12]. Therefore, variation in water forms and overall content was premise for the deterioration of WHC in WB product, while the deterioration of WHC could be classified as the fundamental reason for the decline of chicken patty quality.

We found that 25% inclusion of WB, there was no significant difference in the quality of chicken patties in the areas of degree of lipid and protein oxidation, hardness, sensory parameters, etc., when compared to the control containing no WB. This indicates that WB inclusion in ground meat for chicken patty formulation is feasible. In addition, the small gradient experiment (±5%) expressed that 35% WB inclusion could maintain the quality of chicken patties to some extent. Previous studies have shown adding an appropriate amount of WB can improve the taste of chicken products to an extent. Qin [42] verified on a plant scale that formulations of sausage and two types of chicken nuggets enabled the addition of WB to replace anywhere from 15% to 30% of normal breast meat without causing significant quality changes. According to the results of quality analysis in the present study, the inclusion of 35% WB meat was the maximum amount to maintain overall quality of the chicken patties.

## 5. Conclusions

In this study, low-field NMR was used to analyze differences in the water distribution of chicken patties. Increasing WB inclusion levels increased water freedom in chicken patties, as well as producing a significant shift in the proportion of free and combined water. Lipid and protein oxidation increased with an increase in WB inclusion, which indicates that the processing characteristics of WB are closely related to lipid oxidation, protein conformation, and their individual properties. Meanwhile, a significant deterioration of quality indicators such as FL, color, texture, microstructure, and sensory characteristics was present in patties containing high levels of WB inclusion. These results indicate that differences in water distribution affected the quality of chicken patties to some extent and could be the potential reason for the deterioration of quality in chicken patties. Fortunately, none of the quality characteristics of chicken patties were significantly affected when the WB content was less than 25%. In addition, the extrapolation analysis of some quality characteristics of chicken patties showed that the maximum WB content in chicken patties could be extended to 35%. Considering the differences in chicken deep processing technology and economic benefits in different poultry industries, this study recommends that WB inclusion should not exceed 30% during chicken patty processing. Overall, the introduction of WB as raw meat to explore quality differences of chicken products proved as a feasible option for incorporation into chicken patties. Woody breast inclusion provides some theoretical support for further processed products based and provides a new method to reduce the economic loss caused by the devastating myopathy. However, the nutritional value and digestibility of chicken patties, supplemented with WB, still needs to be investigated further.

## Figures and Tables

**Table 1:** Sensory determination standard for chicken patties.

Parameter	Definition	Grade and Grading Criteria
Character phase	Hardness and cohesiveness of samples, compressed the sample with tooth and feeling of the degree of breakage	Hard, sticky taste (0–25); Soft texture, slightly sticky (26–50); Moderate soft and hard, not sticky (51–75); Entrance is soft and elastic (76–100)
Organization phase	Stomata and section structure of the chicken patties were observed along the inside or cross section of the chicken patties	Internal porosity, loose structure (0–25); More internal pores and complete structure (26–50); A small number of stomata and particulate matter, structural integrity (51–75); No porosity, compact structure (76–100)
Taste phase	Smell (aromatic taste sensation) and flavor of chicken patties.	No chicken flavor with peculiar smell (0–25); Has chicken meat flavor, no peculiar smell (26–50); Tastes good and no peculiar smell (51–75); The chicken has strong flavor, good taste and no peculiar smell (76–100)

**Table 2:** Frying loss and color parameters of cooked chicken patties with different woody breast additions.

Measured Parameters		Woody Breast Additions					*p*
		0%	25%	50%	75%	100%	
FL/%		7.80 ± 0.20 ^c^	9.23 ± 0.20 ^c^	11.33 ± 0.42 ^b^	12.47 ± 0.44 ^ab^	13.43 ± 0.80 ^a^	<0.05
Color							
External	L*	62.48 ± 0.47 ^c^	63.27 ± 0.75 ^bc^	63.85 ± 0.46 ^bc^	65.49 ± 0.39 ^a^	65.67 ± 0.76 ^a^	<0.01
	a*	6.91 ± 0.31 ^a^	6.39 ± 0.18 ^a^	4.55 ± 0.54 ^b^	4.00 ± 0.35 ^bc^	3.24 ± 0.09 ^c^	<0.01
	b*	38.13 ± 0.74 ^ab^	38.89 ± 0.37 ^a^	36.96 ± 0.40 ^b^	36.68 ± 0.75 ^b^	33.84 ± 0.28 ^c^	<0.01
Internal	L*	77.77 ± 1.03	78.93 ± 0.18	79.13 ± 0.22	78.85 ± 0.20	77.85 ± 0.76	>0.05
	a*	0.33 ± 0.06 ^a^	0.20 ± 0.07 ^a^	0.13 ± 0.06 ^ab^	0.14 ± 0.05 ^ab^	−0.02 ± 0.10 ^b^	<0.05
	b*	14.08 ± 0.12 ^d^	14.86 ± 0.06 ^c^	15.09 ± 0.16 ^bc^	15.27 ± 0.09 ^b^	15.82 ± 0.07 ^a^	<0.01

^a–d^ means not sharing a common letter in the same row are considered significantly different (*p* < 0.05).

**Table 3:** Texture properties and sensory evaluation of chicken patties with different woody breast additions.

Measured Parameters	Woody Breast Additions					*p*
	0%	25%	50%	75%	100%	
TPA						
Hardness/g	3944.84 ± 88.71 ^a^	3445.24 ± 114.62 ^a^	2457.08 ± 123.80 ^b^	2296.87 ± 114.15 ^b^	1637.81 ± 75.68 ^c^	<0.01
Springiness	0.89 ± 0.01 ^ab^	0.90 ± 0.01 ^a^	0.88 ± 0.01 ^bc^	0.88 ± 0.01 ^bc^	0.86 ± 0.01 ^c^	<0.01
Chewiness/g	2458.90 ± 176.73 ^a^	1790.45 ± 87.55 ^b^	1739.33 ± 85.93 ^bc^	1416.00 ± 88.80 ^cd^	1348.76 ± 125.44 ^d^	<0.01
Cohesiveness	0.73 ± 0.01	0.74 ± 0.01	0.73 ± 0.01	0.74 ± 0.01	0.73 ± 0.01	>0.05
Resilience	0.36 ± 0.01	0.36 ± 0.00	0.37 ± 0.01	0.37 ± 0.01	0.38 ± 0.01	>0.05
Sensory						
Character	56.50 ± 3.17 ^ab^	65.10 ± 4.49 ^a^	48.10 ± 5.63 ^bc^	52.50 ± 2.27 ^bc^	41.80 ± 5.51 ^c^	<0.01
Organization	69.60 ± 2.43 ^a^	66.00 ± 2.21 ^ab^	63.90 ± 2.96 ^ab^	58.60 ± 2.70 ^bc^	52.80 ± 2.90 ^c^	<0.01
Taste	60.50 ± 3.61 ^a^	64.00 ± 3.32 ^a^	50.10 ± 2.11 ^b^	56.90 ± 2.79 ^ab^	49.50 ± 3.36 ^b^	<0.01
Total score	61.63 ± 2.19 ^ab^	65.04 ± 2.02 ^a^	53.44 ± 2.17 ^cd^	55.65 ± 1.37 ^bc^	47.41 ± 2.85 ^d^	<0.01

^a–d^ means not sharing a common letter in the same row are considered significantly different (*p* < 0.05).

## Data Availability

Data sharing is not applicable to this article.
